# Tabloid Thoughts on Sleeplessness

**Published:** 1918-12

**Authors:** Edwin F. Bowers

**Affiliations:** New York


### Tabloid Thoughts on Sleeplessness.

EDWIN F. BOWERS, M. D., NEW YORK.

---

Sleeplessness is a result of one or more of any number of different causes.

Eating too much or not enough before going to bed; cold feet; bad air; retiring with too many of the day's problems unsolved; insomniaphobia, or fear of sleeplessness; too much light or too much noise in the sleeping apartment; being uncomfortably couched; nervousness, worry, and excessive mental activity; a blood supply supersaturated with toxic products; too much blood or not enough; eye-strain; or even grave physical defects, as heart trouble, high blood-pressure, deranged digestion, pain, kidney failure—almost any abnormality may cause insomnia.

The cause of this complex and aggravating condition must be sought before any method of treatment can be successfully instituted. For it can be permanently overcome only by removing or correcting the cause.

Now, while animals feast abundantly, and immediately afterward sleep soundly, the average human being has been civilized away from this fine trait. So, to inure sleeping, it may be sound judgment to eschew the late supper, and always allow a sufficient length of time to elapse between eating and sleeping.

Yet, one should not try to sleep if that "all gone" feeling is present, or if there is a superabundance of blood in the brain. A glass of hot malted milk or broth will "kill the craving." Or a light, easily digested lunch will draw the excess blood from the brain, and invite sleep.

If the feet are cold and clammy—or even on general principles—a hot footbath before turning in will soothe and quiet. In cold weather a pair of warm stockings may be worn with advantage.

### PURE AIR THE BEST NARCOTIC.

Nothing conduces to pleasant slumber more effectively than a gentle current of pure air flowing through the sleeping chamber. Naturally, the bed should be protected from direct drafts, and there should be sufficient bedclothes to prevent any uncomfortable or sleep-dispelling sensations from cold, both in winter and in summer.

"Hard" reading or study, or nocturnal planning of the next day's activities should be rigorously avoided.

Fear of sleeplessness should be pooh-poohed—two or three times. This is a splendid soporific; for courage repels the brooding demand of unrest, while fear invites his presence.

We can minimize noise by placing in the ears cotton plugs dipped in vaseline. And it would be money well spent to secure an easy, comfortable bed, devoid of protuberant knobs or projections and divested of the disturing influence of any other sleeper.

### AUTO-INTOXICATION MAY CAUSE SLEEPLESSNESS.

Nervousness resulting from auto-intoxication can best be overcome by regulating the diet—limiting the quantity of meats, eliminating tea, coffee, alcohol, and also tobacco in excess, and by clearing the under-oxidized debris from the system.

We have learned a lot about auto-intoxication in recent years. Many sedentary workers suffer from it without knowing why they suffer. A careful adherence to a sensible diet and regimen will invariably benefit these cases.

If the insomniac is troubled with headaches, usually relieved by a night's rest, it is highly probable that some ocular condition exists. This should be submitted to an oculit for correction.

Almost anything that soothes and quiets is good—except drugs, which should never be used except as a means of "breaking in" on the habit of insomnia.

Treat insomnia with intelligence and discrimination, and it will fold its wings and decamp to fresh fields of mischief.

